# Genus level analysis of PKS-NRPS and NRPS-PKS hybrids reveals their origin in Aspergilli

**DOI:** 10.1186/s12864-019-6114-2

**Published:** 2019-11-13

**Authors:** Sebastian Theobald, Tammi C. Vesth, Mikael R. Andersen

**Affiliations:** 1 Department of Biotechnology and Biomedicine, Technical University of Denmark, Søltofts Plads 223, Kgs. Lyngby, Denmark; 2 Current address: Novozymes A/S, Krogshøjvej 36, Bagsværd, Denmark; 3 Current address: Chr. Hansen, Bøge Alle, Hørsholm, Denmark

**Keywords:** Aspergillus, PKS-NRPS hybrids, Secondary metabolites, Gene clusters

## Abstract

**Background:**

Filamentous fungi produce a vast amount of bioactive secondary metabolites (SMs) synthesized by e.g. hybrid polyketide synthase-nonribosomal peptide synthetase enzymes (PKS-NRPS; NRPS-PKS). While their domain structure suggests a common ancestor with other SM proteins, their evolutionary origin and dynamics in fungi are still unclear. Recent rational engineering approaches highlighted the possibility to reassemble hybrids into chimeras — suggesting molecular recombination as diversifying mechanism.

**Results:**

Phylogenetic analysis of hybrids in 37 species – spanning 9 sections of *Aspergillus* and *Penicillium chrysogenum* – let us describe their dynamics throughout the genus *Aspergillus*. The tree topology indicates that three groups of PKS-NRPS as well as one group of NRPS-PKS hybrids developed independently from each other. Comparison to other SM genes lead to the conclusion that hybrids in Aspergilli have several PKS ancestors; in contrast, hybrids are monophyletic when compared to available NRPS genes — with the exception of a small group of NRPSs. Our analysis also revealed that certain NRPS-likes are derived from NRPSs, suggesting that the NRPS/NRPS-like relationship is dynamic and proteins can diverge from one function to another. An extended phylogenetic analysis including bacterial and fungal taxa revealed multiple ancestors of hybrids. Homologous hybrids are present in all sections which suggests frequent horizontal gene transfer between genera and a finite number of hybrids in fungi.

**Conclusion:**

Phylogenetic distances between hybrids provide us with evidence for their evolution: Large inter-group distances indicate multiple independent events leading to the generation of hybrids, while short intra-group distances of hybrids from different taxonomic sections indicate frequent horizontal gene transfer. Our results are further supported by adding bacterial and fungal genera. Presence of related hybrid genes in all Ascomycetes suggests a frequent horizontal gene transfer between genera and a finite diversity of hybrids — also explaining their scarcity. The provided insights into relations of hybrids and other SM genes will serve in rational design of new hybrid enzymes.

## Background

Secondary metabolites (SMs), non-growth associated compounds, have been subject to research efforts due to their wide range of bioactivities. Polyketides like sterigmatocystin and aflatoxin, two potent mycotoxins [1], cause food spoilage; while others like the cholesterol lowering lovastatins can be used as medical drugs [2]. Many SMs are promising leads for anti-cancer drugs as e.g. the non-ribosomal peptide malformins [3]. The enzyme classes producing these distinct compounds — polyketide synthases (PKSs) and non-ribosomal peptide synthetases (NRPSs) — are also seen in PKS-NRPS or NRPS-PKS hybrids, seemingly chimeric genes creating a chimeric compound. The products of hybrids are often bioactive, e.g. the mycotoxins cyclopiazonic acid, pyranonigrin, and cytochalasin. [4–6].

The evolutionary events leading to new enzymes and hence compounds have been described in detail for PKSs and NRPSs. PKSs diversify by exchange of initiation modules for modification of primer units, module duplication, horizontal gene transfer [7, 8]. Other studies suggest a burst of PKS duplications in the early *Pezizomycotina*, a predecessor of mainly *Ascomycota*, [9] as major driver for PKS diversity. For NRPSs, studies suggest duplication and loss of NRPSs, horizontal gene transfer (HGT) from bacteria to fungi, and gain and loss of domains as driver for diversity [10, 11].

In contrast, hybrids have been neglected by phylogenetic studies, although their combination of NRPS and PKS domains suggests an interesting evolutionary history. The existing studies have focused on known hybrids [12]. Lawrence et al. [13] have shown that one *Cochliobolus heterostrophus* NRPS-PKS hybrid gene originates from Burkholderiales; which they suggest to be acquired by HGT in the early evolution of the Pezizomycotina. A model for the avirulence factor *ACE1* gene [14] by Khaldi et al. [15] shows gene duplication, loss, and horizontal gene transfer — a common event between fungi [16] — as the driver in diversification of *ACE1* hybrids. *ACE1* duplicated in ancestors of *Eurotiomycetes* and *Dothideomycetes* giving rise to an ACE1-like hybrid, then during diversification of species. Within *Aspergillus* species, only *A. clavatus* preserved the ACE1-like hybrid and in addition received ACE1 from *Magnaporthe grisea* through HGT (ccsA).

Using genome data from 38 strains of the SM-rich *Aspergillus* genus and *Penicillium chrysogenum* we describe the phylogenetic dynamics of PKS-NRPS and NRPS-PKS hybrids and relate them to PKSs and the structurally similar PKS-likes, as well as NRPSs and the structurally similar NRPS-likes (often the likes show shorter domain arrangement). The genus *Aspergillus* can be divided in taxonomic sections, i.e. groups of fungi with similar morphological and metabolic characteristics, as e.g. the black Aspergilli of section *Nigri*. Due to their morphology, the species of this section were divided in biseriates and uniseriates, which reflects their metabolic capabilites [17] and genetic diversity [18, 19].

Furthermore, we identify origins of hybrids in bacteria and fungal genera. Understanding hybrid evolution and diversity will provide insights into molecular evolution and put rational engineering of these proteins within our grasp.

## Results

### Genus wide analysis identifies independent groups of hybrids

Recent work has highlighted the dynamics of SM genes in fungi and their diversifying mechanisms [7–11]. In this study, we are describing the diversity of rare PKS-NRPS and NRPS-PKS hybrids and compare them to related classes like PKSs and NRPSs. Due to the similarity of these enzymes, we expect that a fusion of NRPSs and PKSs could have occurred during early fungal evolution.

**Fig. 1:**
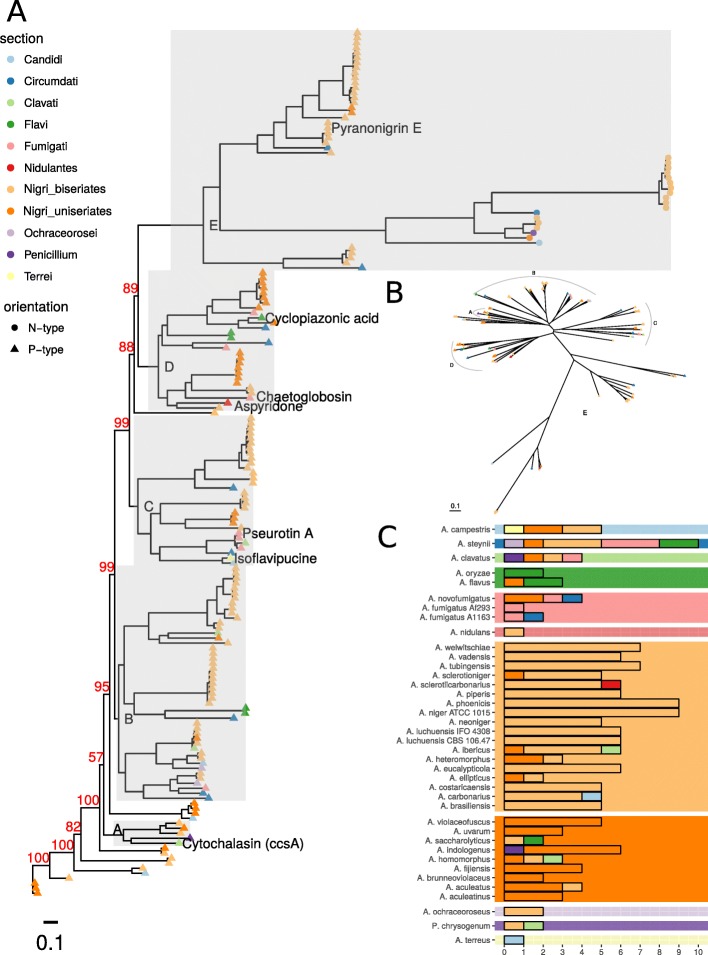
Hybrid dynamics throughout Aspergilli. (**A**) Maximum Likelihood (ML) phylogeny of PKS-NRPS and NRPS-PKS hybrid proteins was created on aligned and trimmed protein sequences. Branches shown in grey (A-E) are shown in Additional file 9: Figure S1, Additional file 10: Figure S2, Additional file 11: Figure S3, Additional file 12: Figure S4, Additional file 13: Figure S5. Sections and species groups are indicated by tip color; the orientation of hybrids N-type (NRPS-PKS) and P-type (PKS-NRPS) is indicated by tip shape. Percentage of 1000 times bootstrap values (red) are indicated for major branches, the remaining values are shown in Additional file 9: Figure S1, Additional file 10: Figure S2, Additional file 11: Figure S3, Additional file 12: Figure S4, Additional file 13: Figure S5. The phylogenetic tree is available as Additional file 1. (**B**) Unrooted view of the phylogenetic tree shown in A. Tip colors indicate sections. Branches highlighted in A are marked in B. (**C**) Classification of nearest neighbors of ML phylogeny. A matrix of tip distances was extracted from the tree and nearest neighbors classified according to their section, thus the barplot shows the origin of hybrid genes by a species. The background color indicates the section of *Aspergillus* species while the bar color indicates the section of the nearest neighbor hybrid ortholog

In order to investigate this, we created a Maximum Likelihood phylogeny (ML) of hybrid proteins from a selection of Aspergilli of section *Nigri* (including biseriates and uniseriates), *Circumdati*, *Candidi*, *Flavi*, *Fumigati*, *Ochracerosii*, *Terrei*, and *P. chrysogenum* to cover several *Eurotiomycetes* (Fig. 1).

In this phylogeny, NRPS-PKS and PKS-NRPS hybrids form several distinct groups, with the PKS-NRPS orientation being more abundant than the NRPS-PKS orientation (Fig. 1, Additional file 1). The analysis indicates related compounds and which hybrids are conserved throughout Aspergilli in different sections.

A group of hybrids containing the cytochalasin producing hybrid *A. clavatus* 6366 (Additional file 9: Figure S1) shows a large phylogenetic distance to other hybrids, indicating that ACE1-like hybrids are rare in Aspergilli — sustaining the hypothesis by Khaldi et al. [15]. Since *A. sclerotioniger* 605326 is the nearest neighbour of *A. clavatus* 6366 we predict this hybrid to produce sclerotionigrin [20], a cytochalasan.

**Fig. 2:**
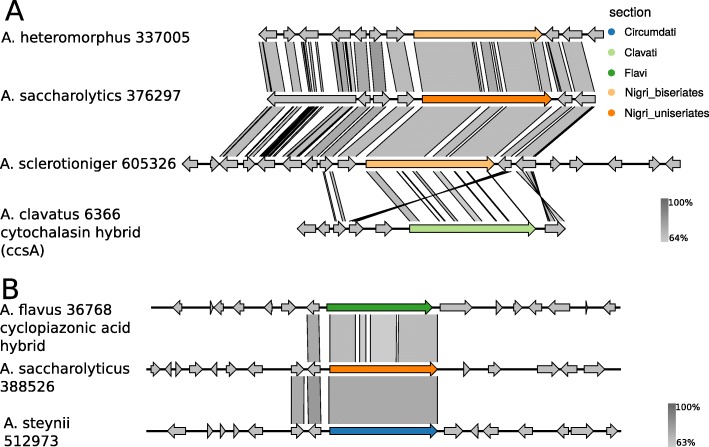
Synteny plots of hybrid gene clusters. **A** Synteny plot of cytochalasin the cytochalasin hybrid from *A. clavatus* and related hybrids from the phylogeny (for phylogeny see Additional file 1). Numbers of species names indicate protein id of the colorized hybrid gene. Color indicates section. *A. sclerotioniger* is known to produce sclerotionigrin — a cytochalasan. The synteny plot shows that the sequence of *A. clavatus* 6366 differs from the other hybrids. **B** Synteny plot of the cyclopiazonic acid hybrid from *A. flavus*. The hybrid gene shows high conservation between Aspergilli from sections Flavi, Nigri and Circumdati (for phylogeny see Additional file 12: Figure S4)

The synteny plot (Fig. 2) further sustains that *A. clavatus* 6366 originates from another genus, since the synteny to related cytochalasan producing hybrids from *Aspergillus* species (e.g. *A. sclerotioniger* 605326) is low.

Another group of hybrids is conserved in biseriate *Nigri* species and *A. homomorphus*, *A. clavatusA. campestris* and *A. ochraceoroseus* (Additional file 10: Figure S2). The tree topology indicates this as common hybrid duplication in Aspergilli through its conservation in many species. The short phylogenetic distance from *A. ibericus* 400692 and *A. sclerotiicarbonarius* 380544 to *A. campestris* 310784 and *A. ochraceoroseus* 492959 hybrids is surprising, as these species are from different sections.

In one case hybrid 370420 of *A. homomorphus* — a member of uniseriate species — is forming a subgroup with hybrids conserved in biseriates of section *Nigri* (Additional file 10: Figure S2). Hence the hybrid has been retained by *A. homomorphus* or gained by horizontal gene transfer (HGT) from another species.

Our results show similar cases to the ACE1 scenario suggested by Khaldi et al. [15]: *A. ibericus* hybrids 443386 and 469268 and *A. steynii* hybrids 454498 and 477231 are duplications with larger phylogenetic distances which suggest duplication, loss, and HGT to happen frequently (Additional file 10: Figure S2).

Hybrids with known compounds show that substrate specificity is unrelated to phylogenetic proximity. Hybrids producing pseurotin A and isoflavipucine (using different substrates) are located in sisterclades (Additional file 11: Figure S3). The broad substrate acceptance of isoflavipucine – shown to create 63 diverse compounds [21] – supports a common origin. Biosynthetically related hybrids like chaetoglobosin and cytochalasin hybrids seem to have evolved in parallel as indicated by the phylogenetic distance in the tree (Additional file 12: Figure S4).

### Sparse hybrids indicate HGT

Following are four subgroups, two consisting of PKS-NRPS, including pyranonigrin related hybrids, and two groups of NRPS-PKS orientation (Additional file 13: Figure S5). Pyranonigrin-related hybrids are, with the exception of hybrid *A. steynii* 463238 unique for section *Nigri* (at least in the scope of our dataset).

Notably, NRPS-PKS hybrids (Additional file 13: Figure S5 indicated by tip labels) are rare among the analyzed species and are only present in a few species: the biseriates of section *Nigri*, *A. indologenus*, *A. steynii*, *A. campestris*, and *Penicillium chrysogenum* (Additional file 13: Figure S5). Their absence in other species and their scarcity point towards recent acquisition by HGT of all NRPS-PKS hybrids.

The phylogeny indicates two major groups of NRPS-PKS hybrids and two hybrids as outgroups (*A. campestris* 323099 and *A. steynii* 418130). While one major group is biseriate specific, the other group consists of *P. chrysogenum* 85311, hybrids from biseriates, and *A. indologenus* 482416. *A. luchuensis* and *A. piperis* are the only species that carry hybrids from both major groups of NRPS-PKS hybrids, pointing towards a HGT before their speciation, or retention of a hybrid. The position of the *P. chrysogenum* 85311 in the phylogeny points towards HGT as well.

### Genus wide analysis provides evidence for HGT

Hybrid diversity in fungi is mostly driven by evolution followed by purifying selection [15] and HGT [13, 15]. With the ML phylogeny established, it was an obvious step to extend our analysis for detection of potential HGT.

If all hybrids were inherited vertically and variation in hybrid content caused by purifying selection, we would expect branches of the phylogenetic tree to only contain hybrids from *Aspergillus* species of the same section and show longer phylogenetic distances due to accumulation of mutations. Hence, identifying homologs of hybrids with short phylogenetic distance from different sections of Aspergilli indicates HGT.

To find the best homologs of hybrids for each species, we extracted distances of hybrids from the ML phylogeny and classified them according to origin (Fig. 1). This analysis works best with the hybrid-rich section *Nigri*, as this group includes many closely related species, which reduce bias, but also the other *Aspergillus* species and *P. chrysogenum* provide insights into hybrid dynamics.

Our analysis reveals that biseriates of section *Nigri* contain mostly conserved groups of hybrids, but can contain some hybrids derived from other sections (Fig. 1 panel C). *A. sclerotiicarbonarius* contains the hybrid 361763 related to the aspyridone hybrid from *A. nidulans* (see also Additional file 11: Figure S3) and *A. heteromorphus* contains a majority of hybrid homologs from uniseriate *Aspergillus* species. *A. ibericus* contains one third of hybrid homologs from other sections. *A. sclerotioniger* 605326 is homolog to hybrid 376297 from uniseriate *A. saccharolyticus*.

Uniseriates of section *Nigri* only contain a low number of hybrid genes, some of them showing orthology to hybrids from other sections (Fig. 1 panel C). *A. saccharolyticus*, a uniseriate, contains one hybrid from biseriate species (376297) and one hybrid (388526) which shows high conservation to a hybrid from *A. steynii* and *A. oryzae*. The latter is responsible for cyclopiazonic acid (CPA) synthesis, a mycotoxin [22].

In this analysis, we included only few non-*Nigri* species with often only one representative per section. Hence, for these cases, Fig. 1 will show that all hybrids in these species have homologs in species of other sections than their own. This is of course biased due to the selection of species, and further genome sequencing in the future will do much to deconvolute this. The analysis does however still give a good indication of the origin of hybrids. E.g. the isoflavipucine hybrid (325) from *A. terreus* and a hybrid (260046) from *A. campestris* show high conservation and thus a short phylogenetic distance which indicates HGT between these species rather than hybrid conservation. Although not derived by HGT, but still worth mentioning, are hybrids from *A. steynii*. Its hybrids are representative of almost every subgroup of hybrids in the dataset, showing a high diversity in this species.

*A. steynii* and section *Nigri* species contain a large number of diverse hybrids which are related to most subgroups of the dataset. If new lineages of hybrids would frequently emerge throughout sections we would expect more section specific hybrids and *A. steynii* as well as *Nigri* species would cover less of the hybrid groups. This suggests that the evolutionary events leading to hybrid generation happened before species diversification in the genus *Aspergillus*. Another observation is that closely related hybrids are present in many phylogenetically distant sections. This points to diversification of hybrids occuring through recombination events after HGT of hybrids.

Additionally, we expect that NRPS-PKS hybrids were either derived by joining of independent NRPS and PKS genes or acquired independently from another source, since they show large phylogenetic distance to PKS-NRPS hybrids. Since the phylogenetic distance could be biased by the amount of structurally similar PKS-NRPS hybrids, we created further comparisons on basis of single domains.

### PKS analysis shows common ancestors for PKSs and hybrids

**Fig. 3:**
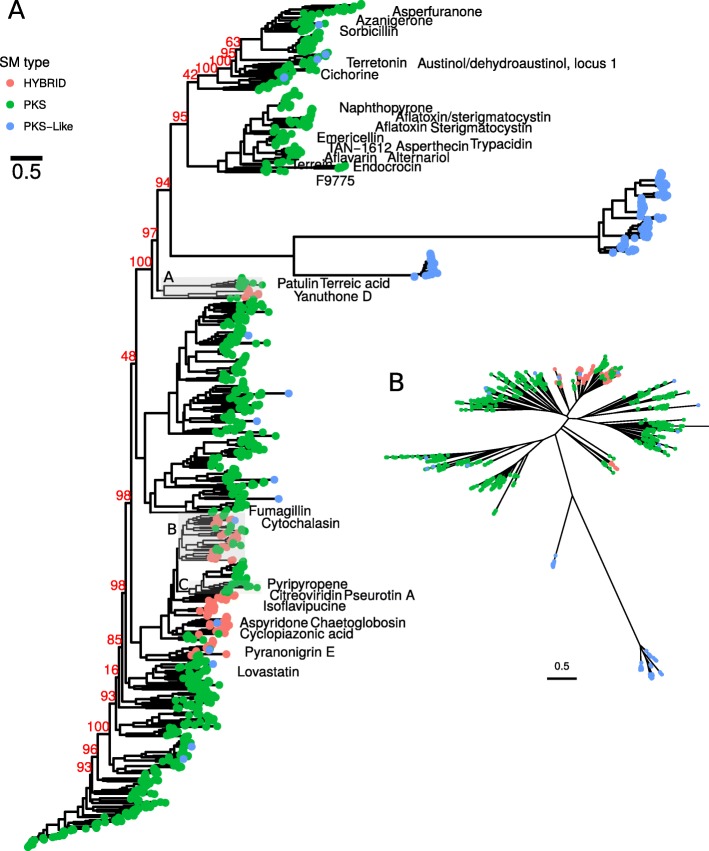
Phylogeny of PKS, PKS-like and hybrid proteins. The maximum likelihood phylogeny was created from KS domains of PKS, PKS-like and hybrid proteins. Tip color shows SM gene type (red: hybrid, green: PKS, blue: PKS-like). Hybrids linked to compounds are labelled with the compound name. Grey highlighted groups A-C are shown in Additional file 14: Figure S6, Additional file 15: Figure S7, Additional file 16: Figure S8. Phylogenetic tree available as Additional file 2

Since intrinsically, hybrids did show large phylogenetic distances (Fig. 1, Additional files 2 and 3), we hypothesized their origin from related SM genes. Previous studies prove hybrid parts as exchangeable [23, 24], hence, we proposed that other SM genes could join together in filamentous fungi to form a hybrid. In order to study this, we created a ML phylogeny of 1369 ketosynthase (KS) domains of PKS-like, PKS and hybrids to elucidate their phylogenetic relations (Fig. 3).

The tree topology shows multiple groups consisting of PKS only, mixed PKS and hybrids and PKS-likes. PKS-likes form two unrelated groups, suggesting that they are largely unrelated to PKSs. Hybrids are separated into two groups. NRPS-PKS hybrids are located as sister clade to 6-methylsalicylic acid (6-MSA) PKS related genes — including PKSs for synthesis of yanuthones, terreic acid and patulin (Fig. 3 branch A, Additional file 14: Figure S6). PKS-NRPSs are clustering together with other PKSs that frequently break into hybrid clades and separate known examples from each other (Fig. 3 branch B, Additional file 15: Figure S7). Thus, we suggest these PKSs and PKS-NRPS hybrids to have common ancestors in fungi. PKSs linked to citreoviridin and pyripyropene are lcoated in a sister clade to hybrids (Fig. 3 branch B, Additional file 16: Figure S8). The pyripyropene PKS has an adjacent adenylation domain in its cluster, thus these PKSs could be the ideal precursor for the molecular evolution of hybrids.

In summary, hybrids do not form a monophyletic clade inside the ML phylogeny — rather, clades contain mixes of PKSs and hybrids. Hence we can hypothesize that PKSs and hybrids had common ancestors — distinct ones for NRPS-PKS and PKS-NRPS genes. Additionally, the analysis shows that NRPS-PKS and PKS-NRPS hybrids are unrelated as indicated by the phylogeny of hybrids (Fig. 1).

### Phylogeny of NRPSs and hybrids reveals monophyletic clade of hybrids

Hybrids incorporate amino acids (e.g. tyrosine in case of the cytotoxic aspyridone or L-phenylalanine in case of cytochalasins) into compounds in a manner similar to NRPS and NRPS-likes. Thus we sought to investigate the phylogenetic relationship of these proteins.

**Fig. 4:**
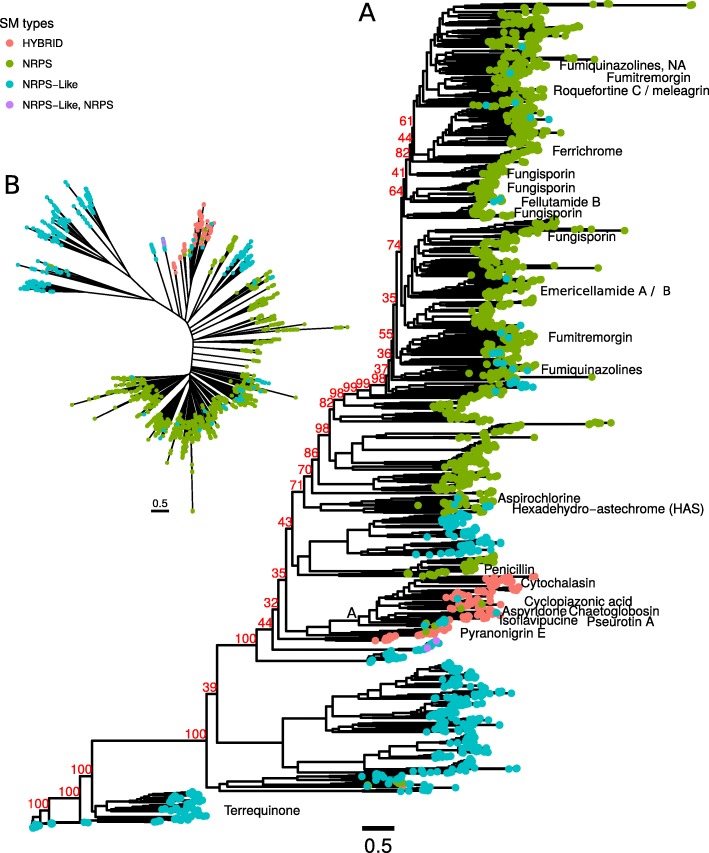
ML phylogeny of NRPS, NRPS-like and hybrid A domains. Tip colors indicate SM protein type; Tip labels show associated compound (if applicable). The phylogenetic tree shows that hybrids are monophyletic when compared to NRPSs and NRPS-likes. Phylogenetic tree available as additional file 3. Branch A shown in Additional file 17: Figure S10

We created a ML tree of 2428 adenylation domains from NRPS, NRPS-like and hybrid proteins which interestingly led to mostly monophyletic groups (Fig. 4, Additional file 4 and 5): NRPS-likes form two groups which are monophyletic. Other groups comprise NRPSs which appear to have a common ancestor, with few NRPS-likes forming a sister clade. This indicates that NRPS-likes developed from NRPSs in certain cases. Hybrids form a monophyletic group, they are however located in a sister clade with a group of NRPS and NRPS-likes conserved in uniseriate *Nigri* species (Additional file 17: Figure S9), NRPS homologs are also found in *A. heteromorphus* and *A. ellipticus*). These proteins could possibly have a common ancestor. Overall, NRPS and hybrid evolution seems to be largely independent. Thus domains seem to be specific for either NRPS or hybrid proteins.

### Extended analysis of hybrids shows two events leading to hybrid evolution

**Fig. 5:**
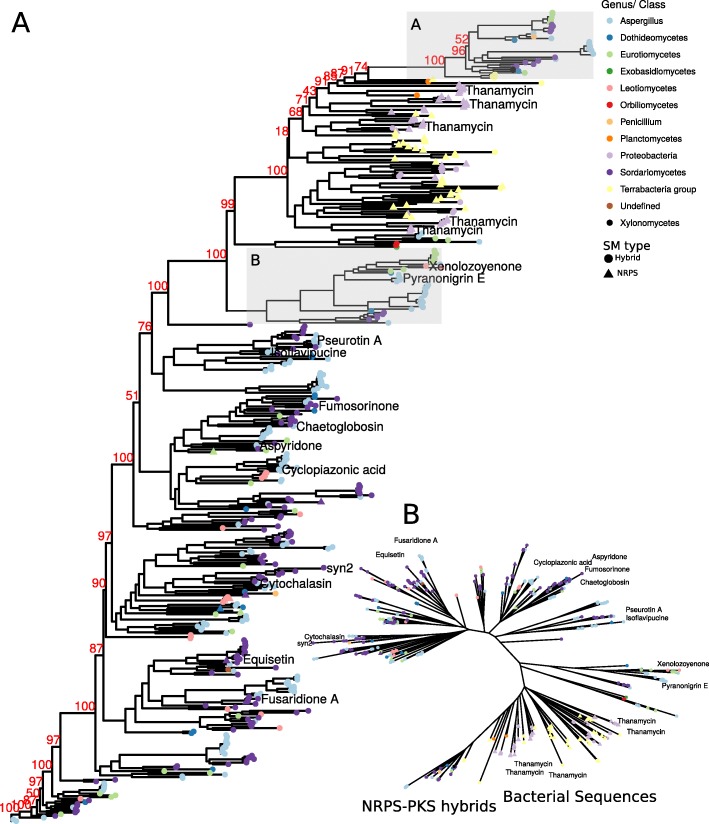
ML phylogeny of A domains from PKS-NRPS and NRPS-PKS hybrid genes and their best blast hits from the NCBI non-redundant protein database. Protein sequences were aligned with clustal omega, trimmed and filtered using trimal, and a ML phylogeny generated using iqtree. Compounds of hybrids, if present in MIBiG are shown above tips. Thanamycin is shown multiple times because each domain of the lipopeptide synthetase is annotated. Tip colors indicate whether the hybrid domain is derived from the genera *Aspergillus*, *Penicillium* or a different taxonomical class. Phylogenetic tree available as additional file 6

We used protein blast on the NCBI non-redundant protein database to find homologs of *Aspergillus* hybrid genes. Adenylation domains from 288 best hits were extracted and added to the *Aspergillus* hybrid adenylation domain set. Subsequent alignment and ML analysis generated the phylogeny in Fig. 5.

Best blast hits were mostly originating from Ascomycete classes: Dothideomycetes, Eurotiomycetes, Leotiomycetes, Sordariomycetes, Xylonomycetes, one Orbilliomycete, and one Exobasidiomycete hybrid were included. Hybrids from bacterial classes include Proteobacteria, Terrabacteria and Planctomycetes.

We found fungal sequences distributed throughout the tree, and although many ascomycete taxa are included, the tree topology indicates that hybrids are conserved throughout these taxa. Certainly our blast search might bias the tree topology. Nonetheless, if PKSs and NRPSs would recombine frequently in fungi, we would expect more intermediates. There are some NRPSs, mostly from Sordariomycetes and Dothideomycetes, which are related to PKS-NRPS hybrids. These could be remnants of ancestral NRPSs which have been donors for hybrids in fungi.

The majority of the tree consists of PKS-NRPS hybrids, while NRPS-PKS hybrids from fungi and bacterial NRPSs and hybrids (from Terrabacteria and Proteobacteria), are co-clustering in one location (Fig. 5 branch A, Additional file 18: Figure S10, Additional files 6, 7, and 8). Inside the cluster, we can identify the thanamycin hybrid gene from *Pseudomonas sp.* SHC52, a lipopeptide. What’s more, we can identify hybrids KPC78190.1, APD71785.1, WP_023586037.1 from *Streptomyces sp.* in a sister clade to hybrids from multiple fungal genera. This indicates that lipopeptides and hybrids from *Streptomyces* could be horizontally transferred to fungi — giving rise to NRPS-PKS hybrids in fungi.

The phylogeny also shows related hybrids of different sections in the same branch, as in the case for genes of aspyridone and fumosorinone — two compounds similar in structure [25]. This supports that the structural diversity of hybrids throughout Ascomycetes might be limited. PKS-NRPS homologs of Aspergilli are co-clustering with many hybrids from Sordariomycetes and Eurotiomycetes. Thus, the distances of pyranonigrin associated hybrids observed earlier (Fig. 1) can now be explained with the added dataset (Fig. 5 branch B, Additional file 19: Figure S11). The *A. ellipticus* hybrid (460246) is clustering closer together with Sordariomycetes; the same for *A. steynii* which carries an Eurotiomycete -related hybrid. Recurrence of the same genera emphasizes that hybrid diversity might be limited in fungi, which is why there are usually so few.

## Discussion

Analysis of the SM protein repertoire of a genus-wide dataset led us to discover dynamics between NRPSs, NRPS-likes, PKSs, PKS-likes and NRPS-PKS as well as PKS-NRPS hybrids. While previous studies included hybrids related to known examples [12], focused on NRPS domains [11], or were considering single hybrids for analysis [13, 15], we combined analysis of A and KS-domains through all hybrids in a genus wide species set and related them to other SM enzymes to focus on their evolutionary history.

Gallo et al. [12] also showed that the hybrid and NRPS adenylation domains are monophyletic — which our analysis supports. Yet, we could identify a sister clade of NRPS and NRPS-likes which groups together with hybrids. These genes are valuable leads in the investigation of SM gene evolution in the genus *Aspergillus*. Additionally, one common ancestor of PKSs and hybrid PKS-NRPSs had been hypothesized [12]. Our results indicate multiple PKSs which could be the ancestor of hybrids. We can confirm that despite structural (or biosynthetic) similarity of cytochalasins and chaetoglobosisn, the genes for their synthetases have a distinct phylogenetic history — *ccsA*, the cytochalasin hybrid, is more closely related to the equisetin hybrid than to the chaetoglobosin hybrid.

Previous studies indicated that NRPS-PKSs are of bacterial origin, while PKS-NRPSs, due to their abundance in fungi, are of fungal origin [13]. Our analysis shows that only the A and KS domains of NRPS-PKS hybrids have similar phylogenetic histories in fungi (as indicated in [13]). The extended comparison to hits from the NCBI non-redundant protein database in this study revealed that the collective of NRPS-PKSs in *Ascomycetes* is related to bacterial hybrids and lipopeptide synthetases, suggesting a bacterial origin. PKS-NRPS hybrids on the other hand, show similarity to different fungal PKSs in the ML phylogeny. Hence, we suspect that A and KS domains have a different phylogenetic history in this type of hybrids. Their monophyly in A domain comparisons suggests that one NRPS ancestor was able to recombine with different PKSs. Hence our analysis indicates that multiple events rather than one event gave rise to hybrid evolution.

According to previous studies, the *ccsA* ancestor was duplicated during Ascomycete evolution, lost in Aspergilli, and reacquired by HGT in *A. clavatus* from *Magnaporthe grisea* [15]. In our analysis of SM genes throughout 38 *Aspergillus* strains — with addition of best hits from the NCBI non-redundant protein database — we can further provide evidence for HGT of hybrids through Ascomycetes. Biseriate species *A. sclerotioniger*, *A. heteromorphus* as well as uniseriate species *A. saccharolyticus*, all of which belong to section *Nigri*, contain conserved *ccsA* homologs (Fig. 1). *A. sclerotioniger* is producer of sclerotionigrin, a SM related to cytochalasins from *A. clavatus*.

Duplication, loss and reacquisition of related hybrids appears to be common since *A. ibericus* and *A. steynii* contain duplications with larger phylogenetic distances (Aspibe1_443386 and Aspibe1_469268; Aspste1_454498 and Aspste1_477231). This duplication, loss, and reacquisition pattern is similar to the one proposed for *ace1*. The *ace1* gene is duplicated during Sordariomycete evolution to *ace1* and *syn2*. In an HGT event, *syn2* is transferred from *Magnaporthe grisea* to *A. clavatus* resulting in *ccsA* — an organism which lost the original *ace1* ancestor [15]. Additionally, we find several cases where phylogenetic distances to hybrids of other sections are shorter than to hybrids of their own section.

For example, *A. heteromorphus*, although biseriate, contains mostly hybrid homologs from uniseriate species and *A. saccharolyticus* contains a homolog to a hybrid from *A. flavus*. This is further supported by our extended analysis using additional hybrids from other fungi and bacteria. *A. ellipticus* and *A. steynii* show homologs to Sordariomycetes and Eurotiomycetes, respectively — indicating a loss and reaquisition of a pyranonigrin-related hybrid. The phylogenetic relationship of hybrids points to few events which yielded novel hybrids, the phylogenetic distances however, exclude a loss only diversification. We suggest that hybrids are frequently transferred, hence the short distances between hybrids of distantly related species and the finite number of clades/their low amount.

Aspergilli show vast amounts of SM genes; hybrids however, are the minority (Table 1). These low counts, their dynamics and their unrelated groups (Fig. 1) pose the question whether independent events lead to their evolution and whether their diversity is finite.

**Table 1:** Number of hybrids and total number of predicted SM proteins in *Aspergillus* species and *Penicillium chrysogenum*

Complete name	Jgi name	Section/Group	Hybrids	Total SM
				proteins
*A. campestris*	Aspcam1	*Candidi*	5	48
*A. steynii*	Aspste1	*Circumdati*	10	98
*A. clavatus*	Aspcl1	*Clavati*	4	43
*A. flavus*	Aspfl1	*Flavi*	3	72
*A. oryzae*	Aspor1	*Flavi*	2	68
*A. fumigatus* A1163	Aspfu_A1163_1	*Fumigati*	2	36
*A. fumigatus* Af293	Aspfu1	*Fumigati*	1	37
*A. novofumigatus*	Aspnov1	*Fumigati*	4	59
*A. nidulans*	Aspnid1	*Nidulantes*	1	63
*A. brasiliensis*	Aspbr1	*Nigri_biseriates*	5	82
*A. carbonarius*	Aspca3	*Nigri_biseriates*	5	65
*A. costaricaensis*	Aspcos1	*Nigri_biseriates*	5	97
*A. ellipticus*	Aspell1	*Nigri_biseriates*	2	72
*A. eucalypticola*	Aspeuc1	*Nigri_biseriates*	6	78
*A. heteromorphus*	Asphet1	*Nigri_biseriates*	3	58
*A. ibericus*	Aspibe1	*Nigri_biseriates*	6	57
*A. luchuensis* CBS 106.47	Aspfo1	*Nigri_biseriates*	6	93
*A. luchuensis* IFO 4308	Aspka1_1	*Nigri_biseriates*	6	88
*A. neoniger*	Aspneo1	*Nigri_biseriates*	5	82
*A. niger* ATCC 1015	Aspni7	*Nigri_biseriates*	9	86
*A. phoenicis*	Aspph1	*Nigri_biseriates*	9	89
*A. piperis*	Asppip1	*Nigri_biseriates*	6	86
*A. sclerotiicarbonarius*	Aspscle1	*Nigri_biseriates*	6	82
*A. sclerotioniger*	Aspscl1	*Nigri_biseriates*	5	75
*A. tubingensis*	Asptu1	*Nigri_biseriates*	7	93
*A. vadensis*	Aspvad1	*Nigri_biseriates*	6	85
*A. welwitschiae*	Aspwel1	*Nigri_biseriates*	7	87
*A. aculeatinus*	Aspacu1	*Nigri_uniseriates*	3	90
*A. aculeatus*	Aspac1	*Nigri_uniseriates*	4	73
*A. brunneoviolaceus*	Aspbru1	*Nigri_uniseriates*	2	85
*A. fijiensis*	Aspfij1	*Nigri_uniseriates*	4	94
*A. homomorphus*	Asphom1	*Nigri_uniseriates*	3	78
*A. indologenus*	Aspind1	*Nigri_uniseriates*	6	92
*A. saccharolyticus*	Aspsac1	*Nigri_uniseriates*	2	52
*A. uvarum*	Aspuva1	*Nigri_uniseriates*	3	76
*A. violaceofuscus*	Aspvio1	*Nigri_uniseriates*	5	79
*A. ochraceoroseus*	Aspoch1	*Ochraceorosei*	2	30
*P. chrysogenum*	Pench1	*Penicillium*	2	50
*A. terreus*	Aspte1	*Terrei*	1	73

SM proteins comprise PKS, PKS-like, NRPS, NRPS-like, Hybrids, dimethylallyltryptophan synthase and terpene cyclases

Hybrids were subject to many engineering efforts since their structure suggested inpendendent units, which could be recombined. The genes *tenS* and *dmbS*, part of the hybrids producing the similar compounds tenellin and desmethylbassianin, respectively, could be fused together and resulted in the production of tenellin A [23]. Nielsen et al. [24] created a fusion of CcsA and Syn2 resulting in niduporthin, a novel chimeric compound. Despite these successful recombinations, creating a chimera of hybrids remained challenging as other studies showed a limitation to recombination efforts [26]. Our study will facilitate further efforts to engineer hybrids since we could show their dynamics inside the genus *Aspergillus* and relate it to other fungal genera. Especially NRPSs and PKSs, which have been identified in this study as hybrid related, might be amenable to modification.

## Conclusion

In this study, we were able to show that several hybrid homologs are shared throughout different sections which points to HGT and frequent gene gain and loss events. What’s more, several PKSs were found together with hybrids in clades, while NRPSs and hybrids are mostly monophyletic. A more extensive search against the non-redundant protein database and subsequent phylogenetic analysis shows that hybrids with different orientation have a separate evolutionary origin, they are frequently subject to HGT between taxa and their diversity in *Ascomycetes* appears to be finite, as shown by the clusters of the phylogenetic tree of hybrids. All in all, this study contributes to our understanding of the events which led to hybrid evolution and provide cases for recombination experiments.

## Methods

### Data collection

Protein sequences and SMURF annotations for *Aspergillus* and *Penicillium* species were downloaded from JGI (http://genome.jgi.doe.gov/).

### Genetic dereplication

Secondary metabolite proteins were annotated with known compounds through BLAST against examples from the Minimum information on biosynthetic gene clusters (MIBiG) database [27].

### Identifying best hits in the NCBI non-redundant protein database

Adenylation domains of hybrids were blasted against the NCBI non-redundant protein database (downloaded from http://ftp://ftp.ncbi.nlm.nih.gov/blast/db/) using protein basic local alignment search tool (BLAST) [28]. The best 15 hits which suffice a query coverage cutoff of 80% were retained. Taxonomic labels for best hits were added using ETE 3 toolkit [29] (Additional file 5).

### Protein domains, alignment and maximum likelihood analysis

InterproScan5 (version 5.22-61.0) [30] was used to identify domains on protein sequences. Domains of the same type which were under 350 amino acids long and less than 100 amino acids apart were merged before proceeding. Sequences were handled using Biopython [31]. Resulting domain sequences were aligned using Clustal Omega [32] and cut using trimal [33]. In case of the full hybrid protein tree, full protein sequences were aligned and trimmed. IQ-tree [34] was used on aligned sequences using Model Finder Plus [35] and 1000 times ultra fast bootstrap [36].

### Finding best homologs in trees

A distance matrix was extracted from the Maximum Likelihood tree using the cophenetic function of the ape package [37] in R [38]. Best homologs were plotted using ggplot2 [39].

### Visualization

Phylogenetic trees were visualized using ggtree [40] in combination with the ape package [37] for unrooted trees and ggstance [41]. Gene clusters were plotted using Easyfig [42].

## Supplementary information

**Additional file 1** ML phylogeny of NRPS-PKS and PKS-NRPS hybrids in Aspergilli.

**Additional file 2** ML phylogeny of KS-domains of PKS, PKS-like and hybrid proteins in Aspergilli.

**Additional file 3** List of collections of identical sequences from Additional file 2.

**Additional file 4** ML phylogeny of A domains of NRPS, NRPS-like and hybrid proteins in Aspergilli.

**Additional file 5** Data frame for A-domain tree in additional file 4.

**Additional file 6** ML phylogeny of *Aspergillus* A-domains of hybrid proteins and hybrid proteins from best hit analysis against the non-redundant protein database.

**Additional file 7** Data frame for A-domain tree in additional file 6.

**Additional file 8** NCBI identifier to taxonomical class translation table.

Additional file 9: Figure S1 Branch A from hybrid maximum likelihood phylogeny (Fig. 1). Sections and species groups indicated by tip color; Orientation of hybrids N-type (NRPS-PKS) and P-type (PKS-NRPS) indicated by tip shape. Tip labels constist of jgi organism name, jgi protein id and associated compound (if applicable). Percentage values of 1000 times bootstrap are indicated in red next to the node.

Additional file 10: Figure S2 Branch B from hybrid maximum likelihood phylogeny (Fig. 1). Sections and species groups indicated by tip color; Orientation of hybrids N-type (NRPS-PKS) and P-type (PKS-NRPS) indicated by tip shape. Tip labels constist of jgi organism name, jgi protein id and associated compound (if applicable). Percentage values of 1000 times bootstrap are indicated in red next to the node.

Additional file 11: Figure S3 Branch C from hybrid maximum likelihood phylogeny (Fig. 1). Sections and species groups indicated by tip color; Orientation of hybrids N-type (NRPS-PKS) and P-type (PKS-NRPS) indicated by tip shape. Tip labels constist of jgi organism name, jgi protein id and associated compound (if applicable). Percentage values of 1000 times bootstrap values are indicated in red next to the node.

Additional file 12: Figure S4 Branch D from hybrid maximum likelihood phylogeny (Fig. 1). Sections and species groups indicated by tip color; Orientation of hybrids N-type (NRPS-PKS) and P-type (PKS-NRPS) indicated by tip shape. Tip labels constist of jgi organism name, jgi protein id and associated compound (if applicable). Percentage values of 1000 times bootstrap are indicated in red next to the node.

Additional file 13: Figure S5 Branch E from hybrid maximum likelihood phylogeny (Fig. 1). Sections and species groups indicated by tip color; Orientation of hybrids N-type (NRPS-PKS) and P-type (PKS-NRPS) indicated by tip shape. Tip labels constist of jgi organism name, jgi protein id and associated compound (if applicable). Percentage values of 1000 times bootstrap are indicated in red next to the node.

Additional file 14: Figure S6 Branch A from phylogeny of PKS, PKS-like and hybrid proteins (Fig. 3). Percentage values of 1000 times bootstrap below 100 are shown in red.

Additional file 15: Figure S7 Branch B from phylogeny of PKS, PKS-like and hybrid proteins (Fig. 3). Percentage values of 1000 times bootstrap below 100 are shown in red.

Additional file 16: Figure S8 Branch C from phylogeny of PKS, PKS-like and hybrid proteins (Fig. 3). Percentage values of 1000 times bootstrap below 100 are shown in red.

Additional file 17: Figure S9 Branch A from phylogeny of NRPS, NRPS-like and hybrid proteins (Fig. 4). Tip color indicates section/subgroup; tip shape indicates SM protein type; tip label shows jgi protein id and associated compound (if applicable). A group of NRPS and NRPS-likes from uniseriate *Nigri* species forms a sister clade to the monophyletic hybrids. Percentage values of 1000 times bootstrap below 100 are shown in red.

Additional file 18: Figure S10 ML phylogeny of fungal and bacterial hybrids. Subtree extracted from Fig. 5. Tip labels show species name and NCBI identifier/ JGI organism and protein identifier. Tip color indicates genus or class; Tip shape indicates SM protein type. Percentage values of 1000 times bootstrap below 100 are shown in red. Hybrids from *Streptomyces* form a sister clade to fungal hybrids NRPS-PKS hybrids, indicating this class from bacterial origin.

Additional file 19: Figure S11 ML phylogeny of pyranonigrin associated hybrids. Subtree extracted from Fig. 6. Tip labels show species name and NCBI identifier/ jgi organism and protein identifier. Tip color indicates genus or class; Tip shape indicates SM protein type. Percentage values of 1000 times bootstrap below 100 are shown in red. Additional tip label shows associated compound (if applicable).
