# Supplementary material for: Genus level analysis of PKS-NRPS and NRPS-PKS hybrids reveals their origin in Aspergilli
Source: BMC Genomics. 2019 Nov 13;20:847. doi: 10.1186/s12864-019-6114-2 (PMC6854747; doi:10.1186/s12864-019-6114-2)
Supplement: Supplementary file 9 — Branch A from hybrid maximum likelihood phylogeny (Fig. 1). Sections and species groups indicated by tip color; Orientation of hybrids N-type (NRPS-PKS) and P-type (PKS-NRPS) indicated by tip shape. Tip labels constist of jgi organism name, jgi protein id and associated compound (if applicable). Percentage values of 1000 times bootstrap are indicated in red next to the node. [file 12864_2019_6114_MOESM9_ESM.pdf]

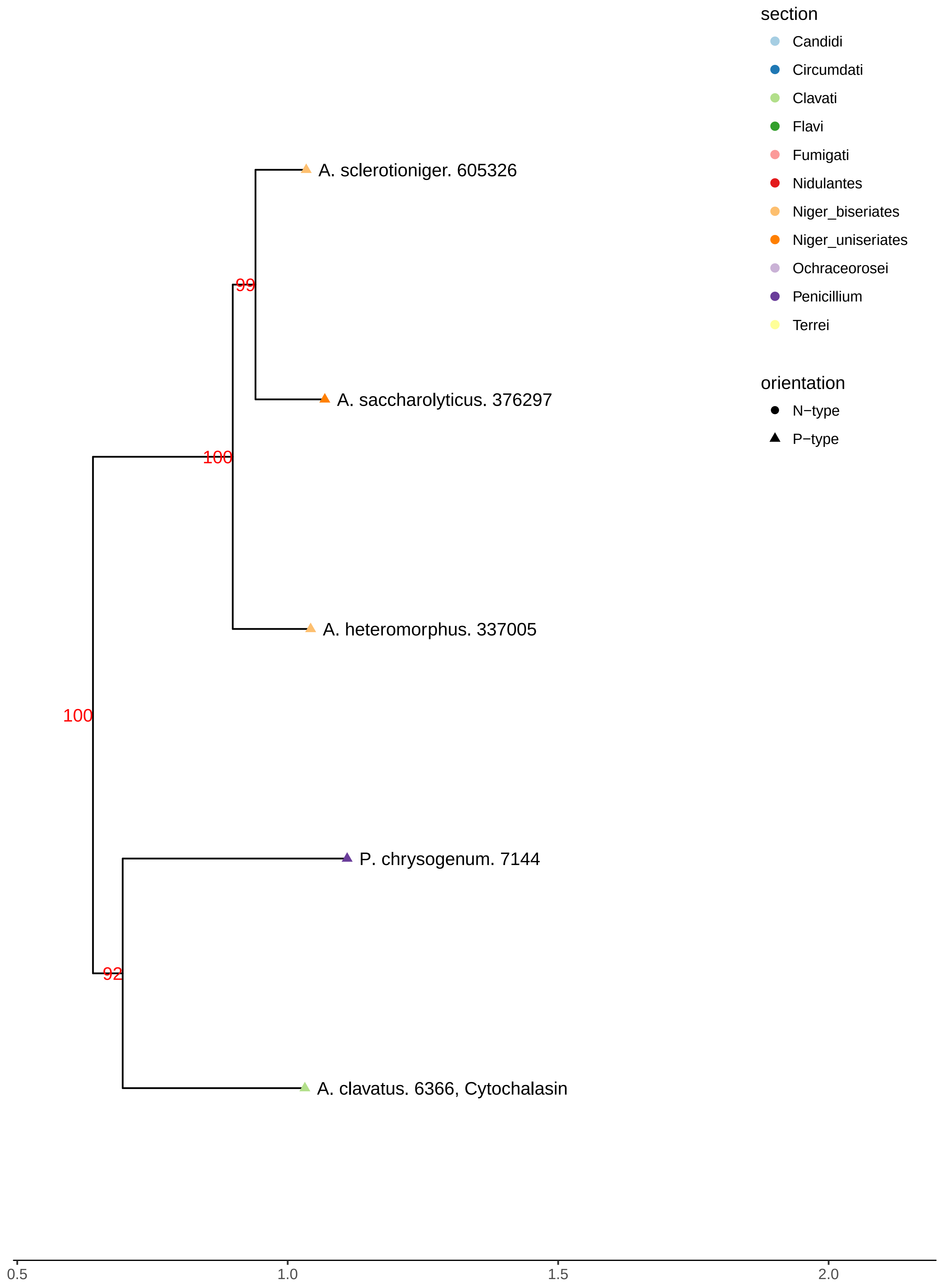
A. sclerotioniger. 605326
A. saccharolyticus. 376297
A. heteromorphus. 337005
P. chrysogenum. 7144
A. clavatus. 6366, Cytochalasin
99
100
100
92
0.5
1.0
1.5
2.0
section
Candidi
Circumdati
Clavati
Flavi
Fumigati
Nidulantes
Niger_biseriates
Niger_uniseriates
Ochraceorosei
Penicillium
Terrei
orientation
N−type
P−type
