# Supplementary material for: Genus level analysis of PKS-NRPS and NRPS-PKS hybrids reveals their origin in Aspergilli
Source: BMC Genomics. 2019 Nov 13;20:847. doi: 10.1186/s12864-019-6114-2 (PMC6854747; doi:10.1186/s12864-019-6114-2)
Supplement: Supplementary file 10 — Branch B from hybrid maximum likelihood phylogeny (Fig. 1). Sections and species groups indicated by tip color; Orientation of hybrids N-type (NRPS-PKS) and P-type (PKS-NRPS) indicated by tip shape. Tip labels constist of jgi organism name, jgi protein id and associated compound (if applicable). Percentage values of 1000 times bootstrap are indicated in red next to the node. [file 12864_2019_6114_MOESM10_ESM.pdf]

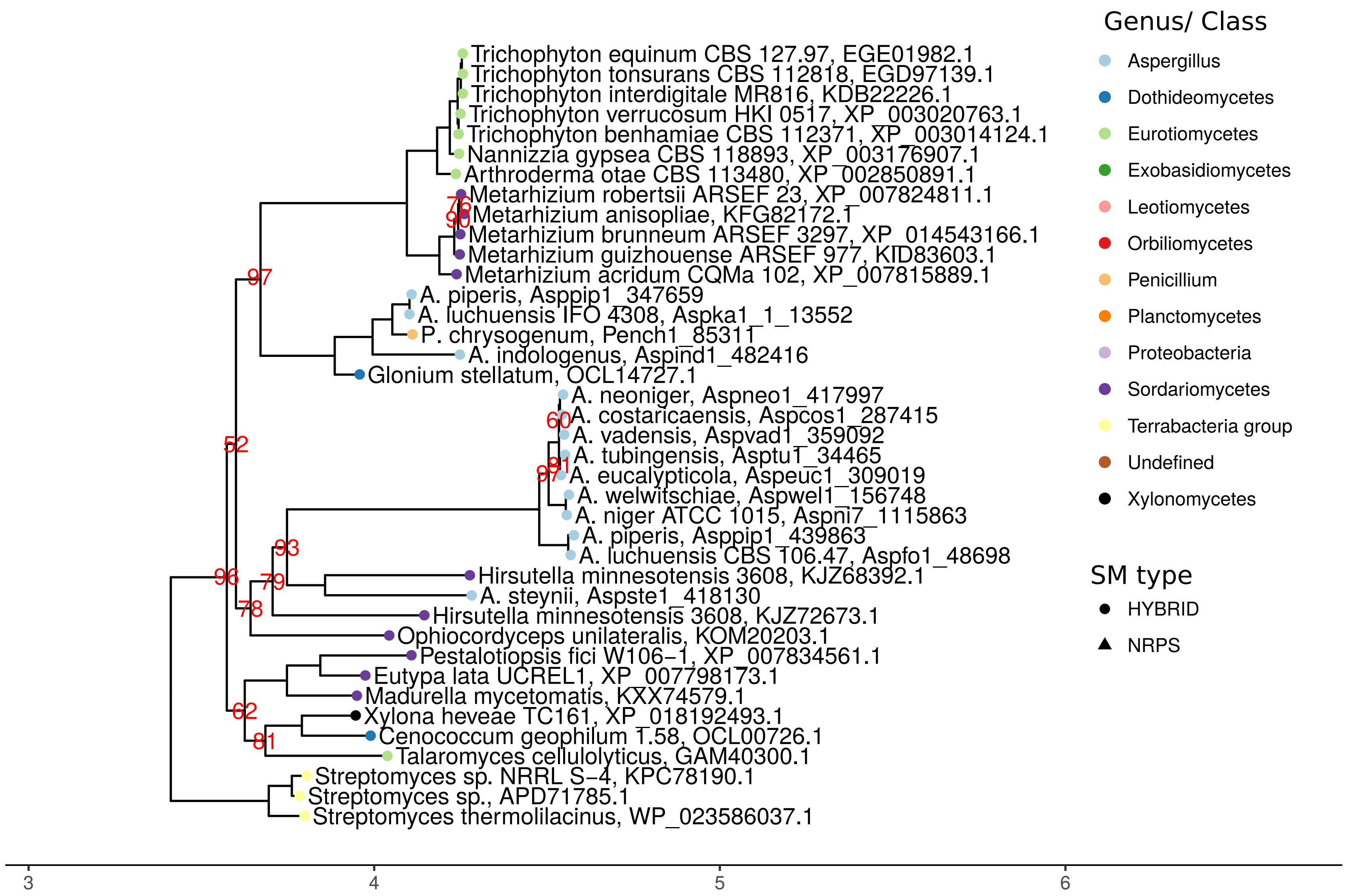
Genus/ Class
Aspergillus
Dothideomycetes
Eurotiomycetes
Exobasidiomycetes
Leotiomycetes
Orbiliomycetes
Penicillium
Planctomycetes
Proteobacteria
Sordariomycetes
Terrabacteria group
Undefined
Xylonomycetes
SM type
HYBRID
NRPS
Trichophyton equinum CBS 127.97, EGE01982.1
Trichophyton tonsurans CBS 112818, EGD97139.1
Trichophyton interdigitale MR816, KDB22226.1
Trichophyton verrucosum HKI 0517, XP_003020763.1
Trichophyton benhamiae CBS 112371, XP_003014124.1
Nannizzia gypsea CBS 118893, XP_003176907.1
Arthroderma otae CBS 113480, XP_002850891.1
Metarhizium robertsii ARSEF 23, XP_007824811.1
Metarhizium anisopliae, KFG82172.1
Metarhizium brunneum ARSEF 3297, XP_014543166.1
Metarhizium guizhouense ARSEF 977, KID83603.1
Metarhizium acridum CQMa 102, XP_007815889.1
A. piperis, Asppip1_347659
A. luchuensis IFO 4308, Aspka1_1_13552
P. chrysogenum, Pench1_85311
A. indologenus, Aspind1_482416
Glonium stellatum, OCL14727.1
A. neoniger, Aspneo1_417997
A. costaricaensis, Aspcos1_287415
A. vadensis, Aspvad1_359092
A. tubingensis, Asptu1_34465
A. eucalypticola, Aspeuc1_309019
A. welwitschiae, Aspwel1_156748
A. niger ATCC 1015, Aspni7_1115863
A. piperis, Asppip1_439863
A. luchuensis CBS 106.47, Aspfo1_48698
Hirsutella minnesotensis 3608, KJZ68392.1
A. steynii, Aspste1_418130
Hirsutella minnesotensis 3608, KJZ72673.1
Ophiocordyceps unilateralis, KOM20203.1
Pestalotiopsis fici W106−1, XP_007834561.1
Eutypa lata UCREL1, XP_007798173.1
Madurella mycetomatis, KXX74579.1
Xylona heveae TC161, XP_018192493.1
Cenococcum geophilum 1.58, OCL00726.1
Talaromyces cellulolyticus, GAM40300.1
Streptomyces sp. NRRL S−4, KPC78190.1
Streptomyces sp., APD71785.1
Streptomyces thermolilacinus, WP_023586037.1
76
90
97
60
81
97
52
93
96
79
78
62
81
3
4
5
6
