# Supplementary figures and images for: Genus level analysis of PKS-NRPS and NRPS-PKS hybrids reveals their origin in Aspergilli
Source: BMC Genomics. 2019 Nov 13;20:847. doi: 10.1186/s12864-019-6114-2 (PMC6854747; doi:10.1186/s12864-019-6114-2)

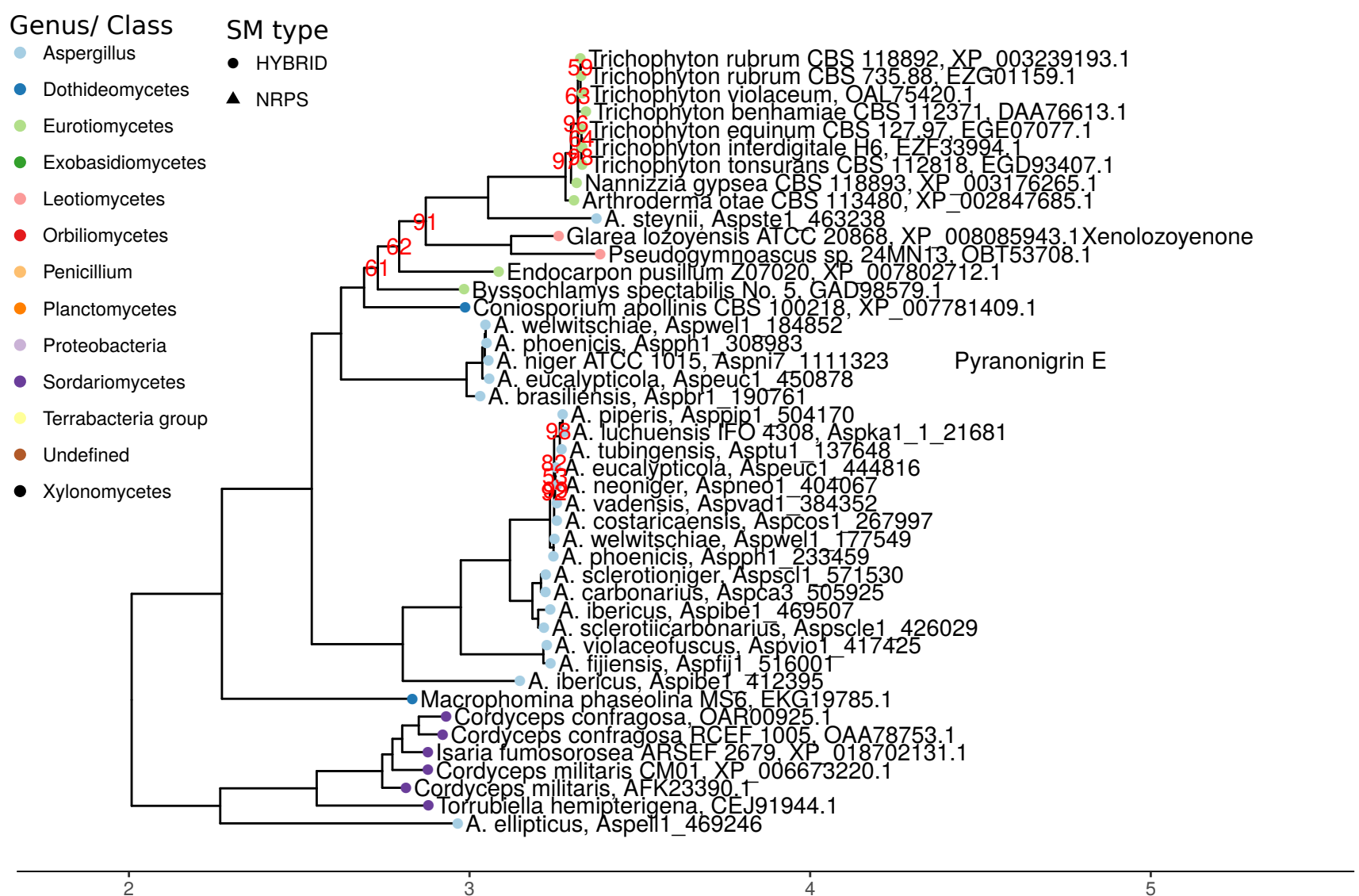

Supplement: Supplementary file 11 — Branch C from hybrid maximum likelihood phylogeny (Fig. 1). Sections and species groups indicated by tip color; Orientation of hybrids N-type (NRPS-PKS) and P-type (PKS-NRPS) indicated by tip shape. Tip labels constist of jgi organism name, jgi protein id and associated compound (if applicable). Percentage values of 1000 times bootstrap values are indicated in red next to the node. [file 12864_2019_6114_MOESM11_ESM.pdf]

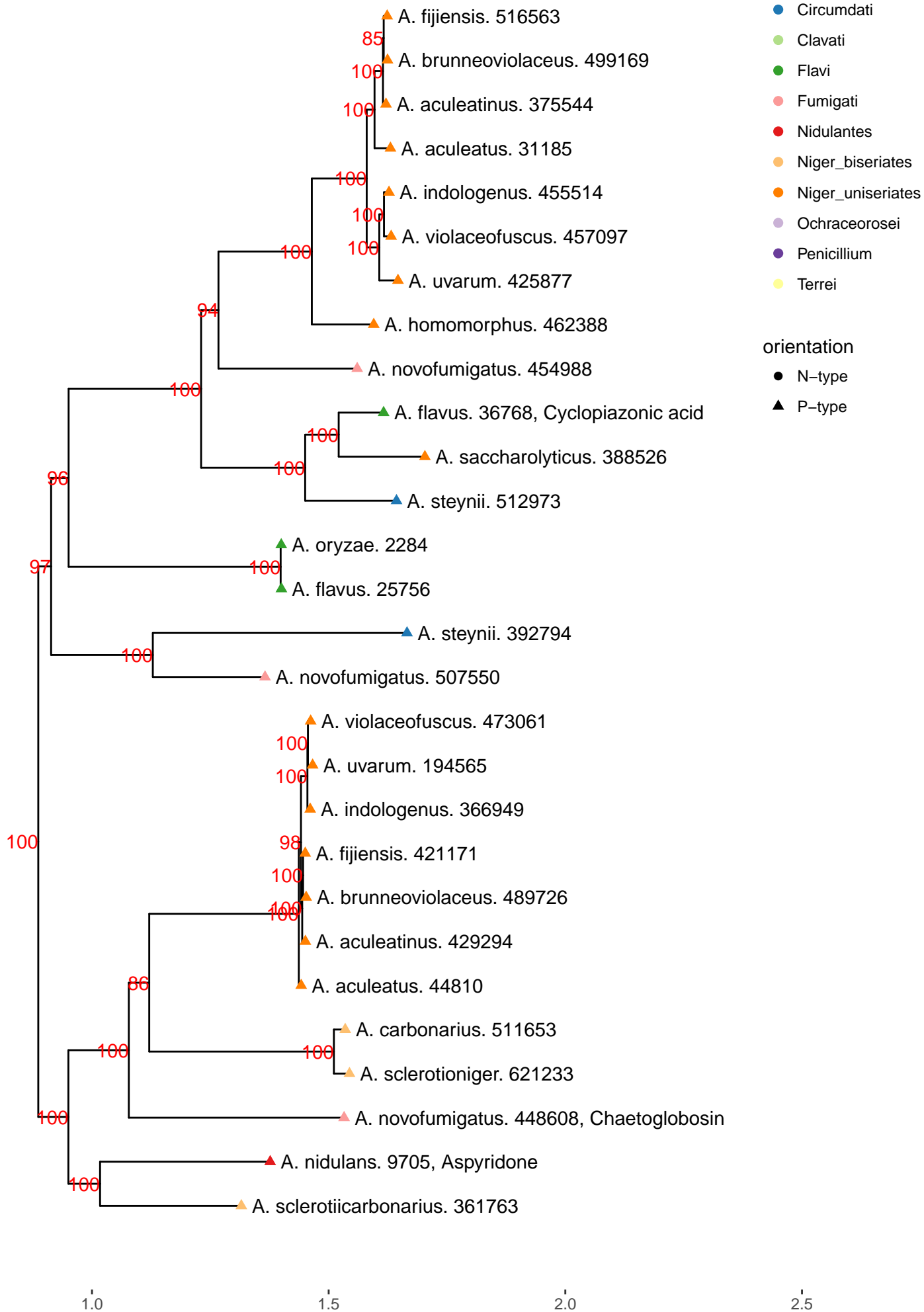

Supplement: Supplementary file 14 — Branch A from phylogeny of PKS, PKS-like and hybrid proteins (Fig. 3). Percentage values of 1000 times bootstrap below 100 are shown in red. [file 12864_2019_6114_MOESM14_ESM.pdf]

SM type

- HYBRID
- PKS
- PKS-Like

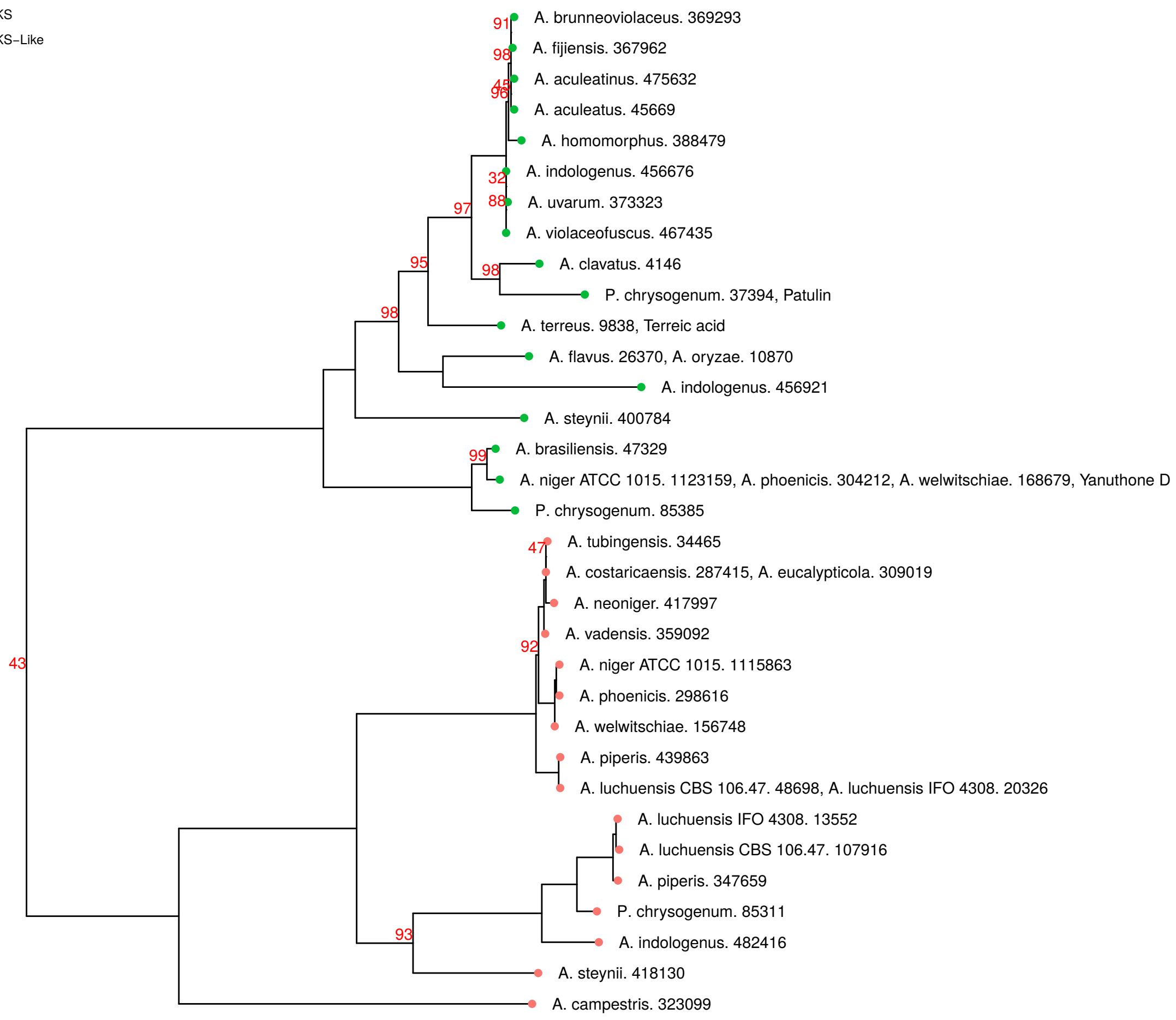

Supplement: Supplementary file 16 — Branch C from phylogeny of PKS, PKS-like and hybrid proteins (Fig. 3). Percentage values of 1000 times bootstrap below 100 are shown in red. [file 12864_2019_6114_MOESM16_ESM.pdf]

SM type

- HYBRID
- PKS
- PKS-Like

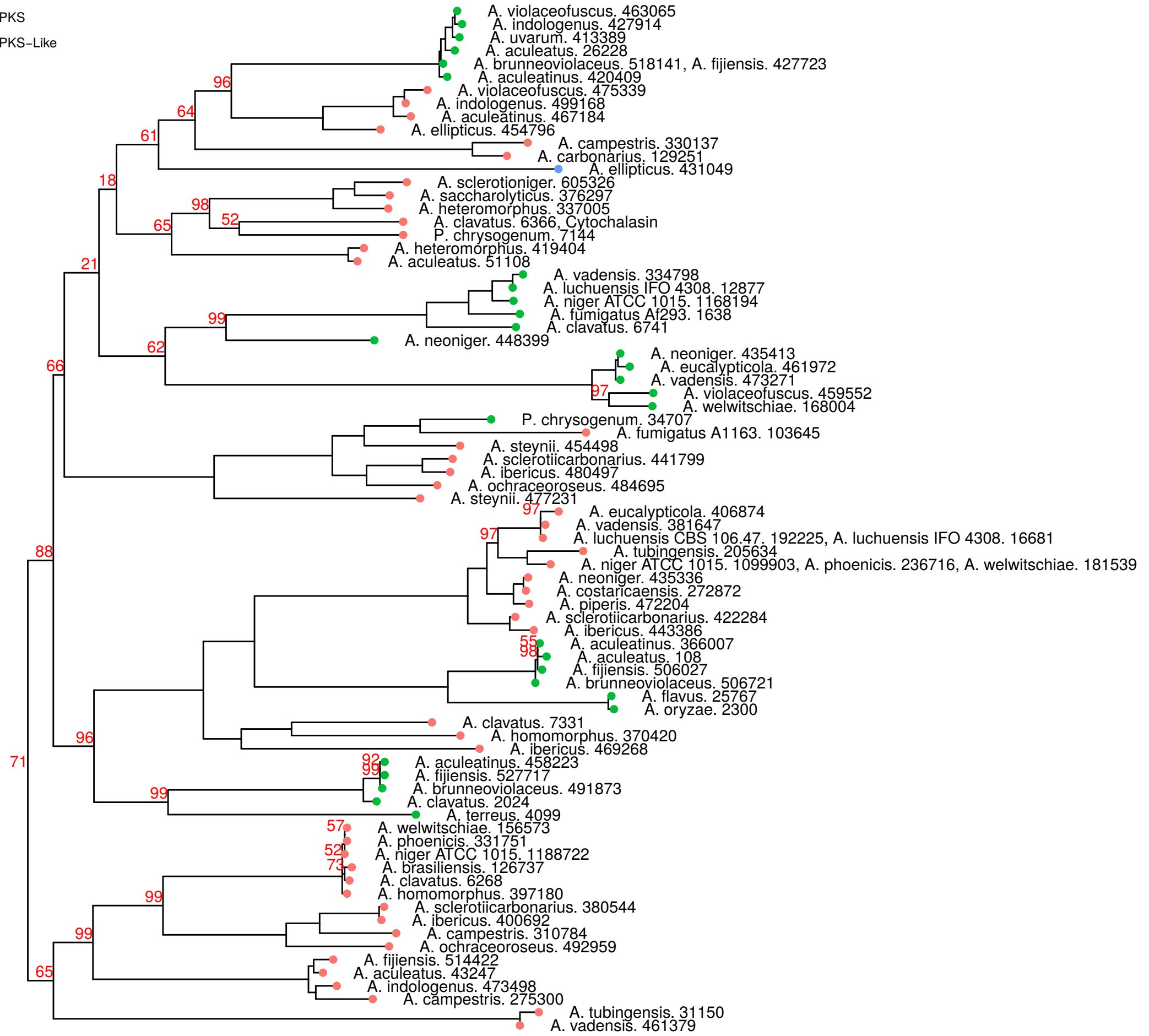

Supplement: Supplementary file 17 — Branch A from phylogeny of NRPS, NRPS-like and hybrid proteins (Fig. 4). Tip color indicates section/subgroup; tip shape indicates SM protein type; tip label shows jgi protein id and associated compound (if applicable). A group of NRPS and NRPS-likes from uniseriate Nigri species forms a sister clade to the monophyletic hybrids. Percentage values of 1000 times bootstrap below 100 are shown in red. [file 12864_2019_6114_MOESM17_ESM.pdf]

SM type

- HYBRID
- PKS
- PKS-Like

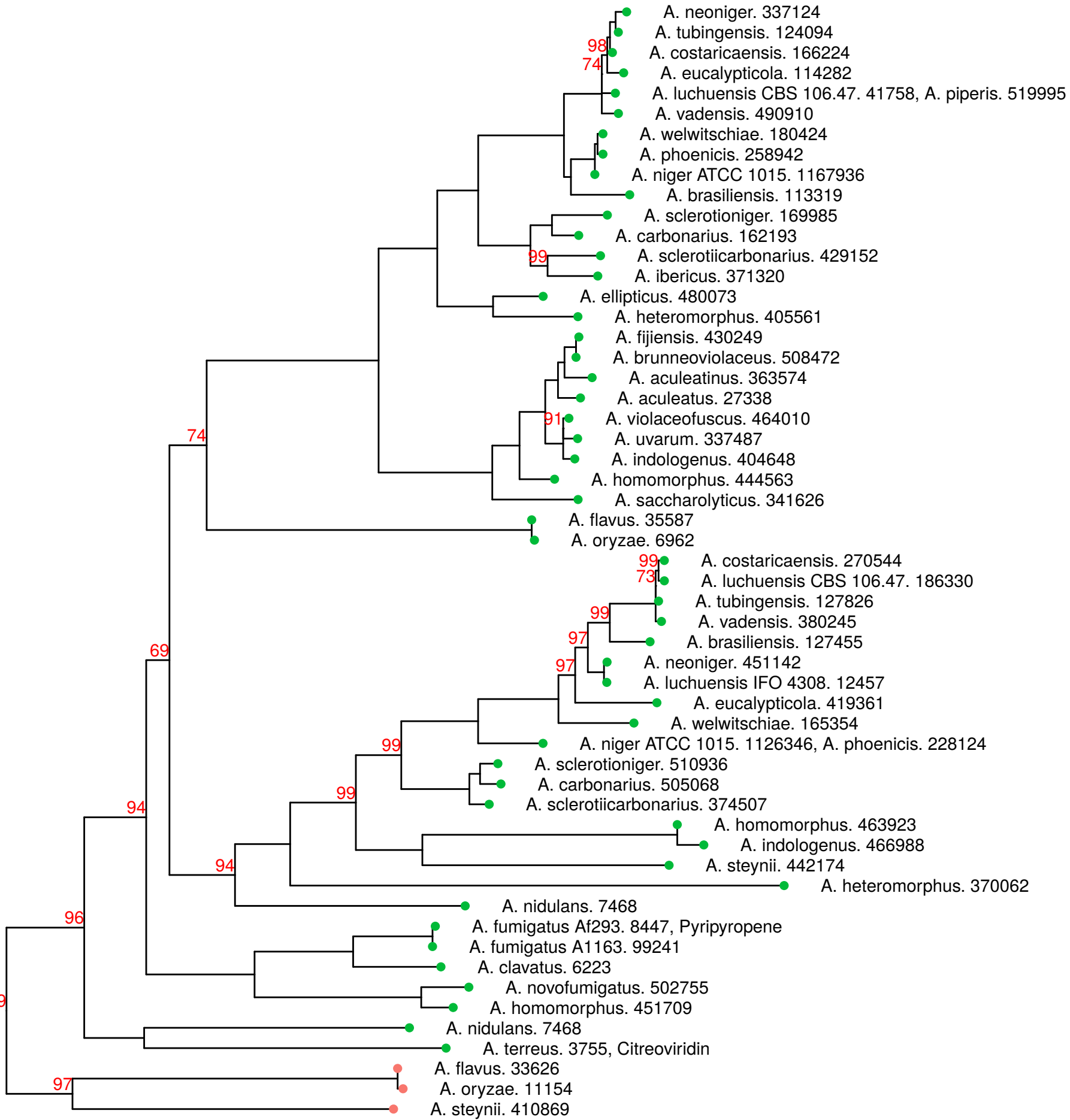

Supplement: Supplementary file 18 — ML phylogeny of fungal and bacterial hybrids. Subtree extracted from Fig. 5. Tip labels show species name and NCBI identifier/ JGI organism and protein identifier. Tip color indicates genus or class; Tip shape indicates SM protein type. Percentage values of 1000 times bootstrap below 100 are shown in red. Hybrids from Streptomyces form a sister clade to fungal hybrids NRPS-PKS hybrids, indicating this class from bacterial origin. [file 12864_2019_6114_MOESM18_ESM.pdf]

- SM type
- HYBRID
  - NRPS
  - NRPS-Like
  - NRPS-Like, NRPS

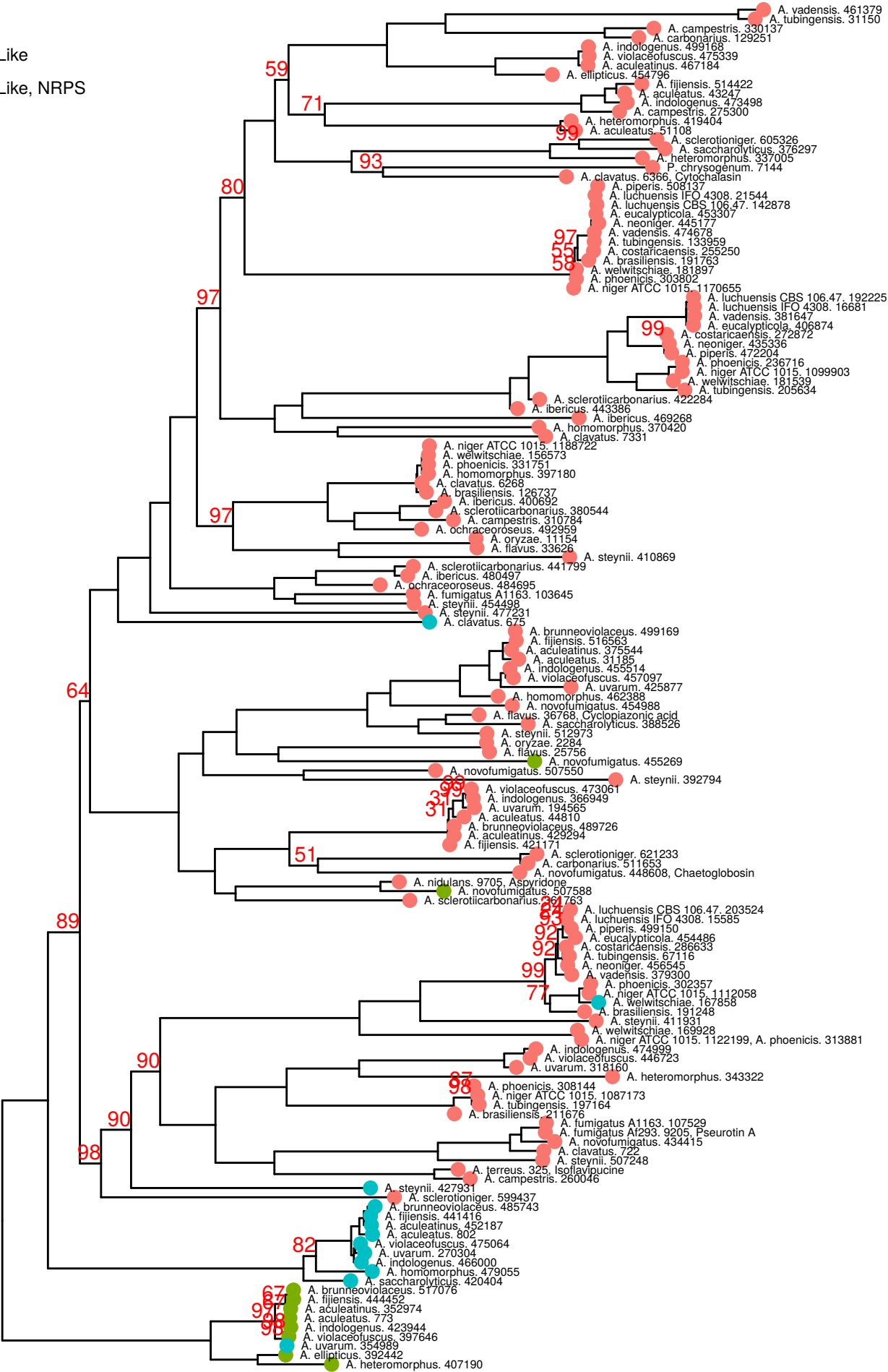

Supplement: Supplementary file 19 — ML phylogeny of pyranonigrin associated hybrids. Subtree extracted from Fig. 6. Tip labels show species name and NCBI identifier/ jgi organism and protein identifier. Tip color indicates genus or class; Tip shape indicates SM protein type. Percentage values of 1000 times bootstrap below 100 are shown in red. Additional tip label shows associated compound (if applicable). [file 12864_2019_6114_MOESM19_ESM.pdf]
