# Supplementary material for: Genus level analysis of PKS-NRPS and NRPS-PKS hybrids reveals their origin in Aspergilli
Source: BMC Genomics. 2019 Nov 13;20:847. doi: 10.1186/s12864-019-6114-2 (PMC6854747; doi:10.1186/s12864-019-6114-2)
Supplement: Supplementary file 15 — Branch B from phylogeny of PKS, PKS-like and hybrid proteins (Fig. 3). Percentage values of 1000 times bootstrap below 100 are shown in red. [file 12864_2019_6114_MOESM15_ESM.pdf]

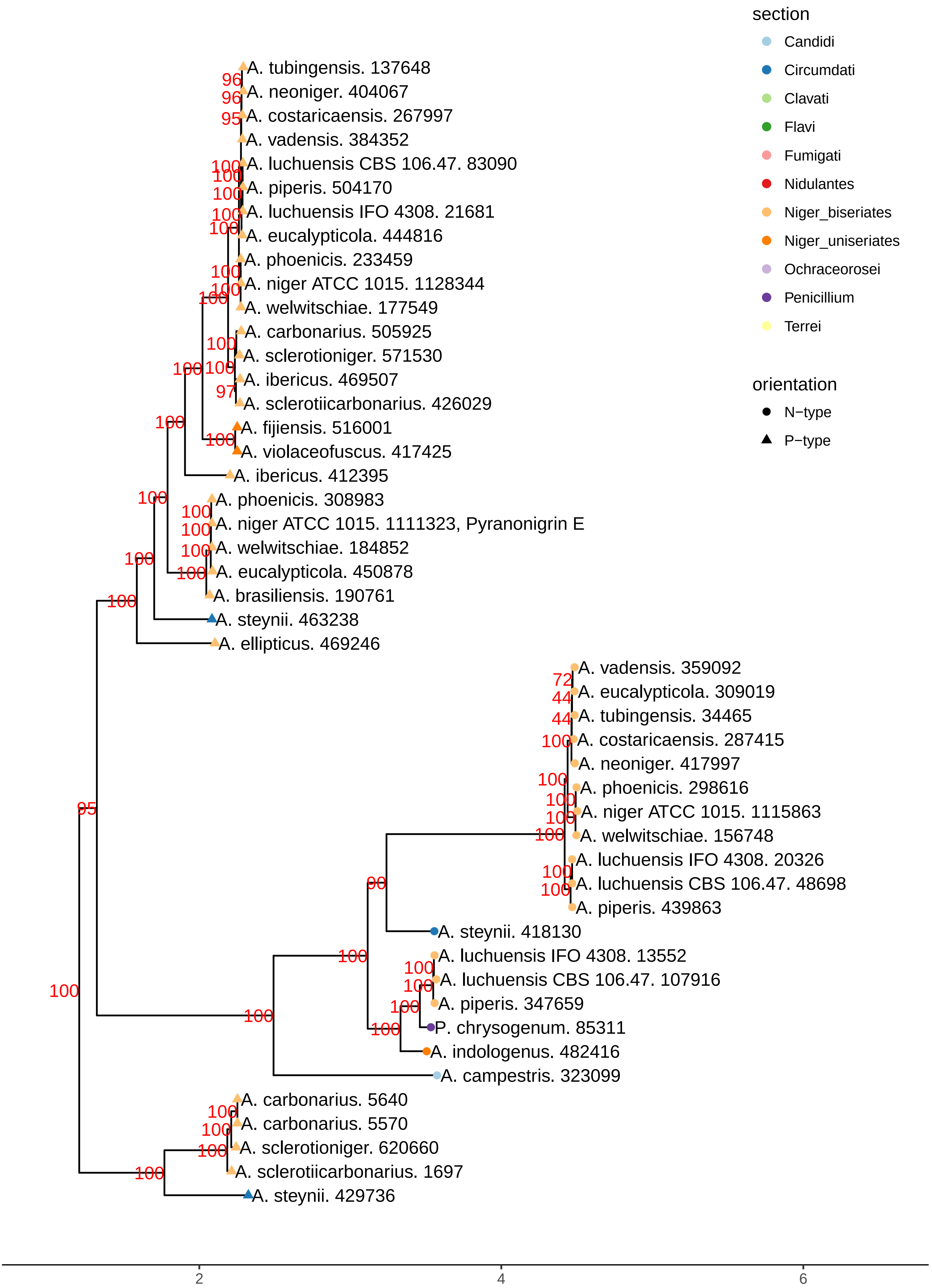
A. tubingensis. 137648
A. neoniger. 404067
A. costaricaensis. 267997
A. vadensis. 384352
A. luchuensis CBS 106.47. 83090
A. piperis. 504170
A. luchuensis IFO 4308. 21681
A. eucalypticola. 444816
A. phoenicis. 233459
A. niger ATCC 1015. 1128344
A. welwitschiae. 177549
A. carbonarius. 505925
A. sclerotioniger. 571530
A. ibericus. 469507
A. sclerotiicarbonarius. 426029
A. fijiensis. 516001
A. violaceofuscus. 417425
A. ibericus. 412395
A. phoenicis. 308983
A. niger ATCC 1015. 1111323, Pyranonigrin E
A. welwitschiae. 184852
A. eucalypticola. 450878
A. brasiliensis. 190761
A. steynii. 463238
A. ellipticus. 469246
A. vadensis. 359092
A. eucalypticola. 309019
A. tubingensis. 34465
A. costaricaensis. 287415
A. neoniger. 417997
A. phoenicis. 298616
A. niger ATCC 1015. 1115863
A. welwitschiae. 156748
A. luchuensis IFO 4308. 20326
A. luchuensis CBS 106.47. 48698
A. piperis. 439863
A. steynii. 418130
A. luchuensis IFO 4308. 13552
A. luchuensis CBS 106.47. 107916
A. piperis. 347659
P. chrysogenum. 85311
A. indologenus. 482416
A. campestris. 323099
A. carbonarius. 5640
A. carbonarius. 5570
A. sclerotioniger. 620660
A. sclerotiicarbonarius. 1697
A. steynii. 429736
section
Candidi
Circumdati
Clavati
Flavi
Fumigati
Nidulantes
Niger_biseriates
Niger_uniseriates
Ochraceorosei
Penicillium
Terrei
orientation
N−type
P−type
2
4
6
